# Familial Hypercholesterolemia and Elevated Lipoprotein(a): Cascade Testing and Other Implications for Contextual Models of Care

**DOI:** 10.3389/fgene.2022.905941

**Published:** 2022-04-27

**Authors:** Wann Jia Loh, Dick C. Chan, Pedro Mata, Gerald F. Watts

**Affiliations:** ^1^ Department of Endocrinology, Changi General Hospital, Singapore, Singapore; ^2^ Medical School, University of Western Australia, Perth, WA, Australia; ^3^ Fundación Hipercolesterolemia Familiar, Madrid, Spain; ^4^ Lipid Disorders Clinic, Department of Cardiology and Internal Medicine, Royal Perth Hospital, Perth, WA, Australia

**Keywords:** cascade testing, familial hypercholesterolemia, lipoprotein (a), Lp(a), inherited hypercholesterolemia, hyper-Lp(a), FH model of care

## Abstract

Elevated lipoprotein(a) [Lp(a)], a predominantly genetic disorder, is a causal risk factor for atherosclerotic cardiovascular disease (ASCVD) and calcific aortic valvular disease, particularly in patients with familial hypercholesterolemia (FH), a Tier I genomic condition. The combination from birth of the cumulative exposure to elevated plasma concentrations of both Lp(a) and low-density lipoprotein is particularly detrimental and explains the enhanced morbidity and mortality risk observed in patients with both conditions. An excellent opportunity to identify at-risk patients with hyper-Lp(a) at increased risk of ASCVD is to test for hyper-Lp(a) during cascade testing for FH. With probands having FH and hyper-Lp(a), the yield of detection of hyper-Lp(a) is 1 individual for every 2.1–2.4 relatives tested, whereas the yield of detection of both conditions is 1 individual for every 3–3.4 relatives tested. In this article, we discuss the incorporation of assessment of Lp(a) in the cascade testing in FH as a feasible and crucial part of models of care for FH. We also propose a simple management tool to help physicians identify and manage elevated Lp(a) in FH, with implications for the care of Lp(a) beyond FH, noting that the clinical use of RNA therapeutics for specifically targeting the overproduction of Lp(a) in at risk patients is still under investigation.

## Introduction

A century ago, a few case reports of patients with sudden cardiac death and xanthomata by Dr. Francis Harbitz, led to the discovery of a heritable condition, that in the words of Dr. Carl Müller was “inherited as a pronounced dominant quality” (Müller, 1938; Goldstein and Brown, 2009). The condition is now known as Familial Hypercholesterolemia (FH). In the 1970s, Joseph Goldstein and Michael Brown discovered the low density lipoprotein (LDL) receptor and the concept of receptor-mediated endocytosis, which explained that FH was due to defective or deficient LDL receptor function (Goldstein and Brown, 1974).

Another clinically important lipoprotein disorder that is under strong genetic influence is elevated plasma lipoprotein(a) [Lp(a)] concentration. Lp(a) was first discovered in 1963 by Kare Berg as an antigenic variant of LDL (Berg, 1963). Large epidemiological, Mendelian randomization, and genome-wide association studies have shown that elevated levels of Lp(a) increase the risk of atherosclerotic cardiovascular disease (ASCVD) and calcific aortic valve disease (CAVD) (Kamstrup et al., 2008; Clarke et al., 2009; Emerging Risk Factors et al., 2009; Kamstrup et al., 2009; Kamstrup et al., 2014; Burgess et al., 2018; Langsted et al., 2019; Perrot et al., 2019; Trinder et al., 2020a). Lp(a) is an LDL-like lipoprotein consisting of a single apolipoprotein B-100 (apoB) covalently bound to apolipoprotein(a) [apo(a)] (Schmidt et al., 2016), a preferential carrier of oxidized phospholipids that also has plasminogen-like properties (Boffa and Koschinsky, 2019). Lp(a) particles accordingly have proatherogenic, prothrombotic, and proinflammatory properties (Nordestgaard et al., 2010; Boffa and Koschinsky, 2019).

The ASCVD risk associated with hyper-Lp(a) is accentuated in patients at high-risk of ASCVD, especially FH. Hence, recommendations on measuring Lp(a) in high-risk patient groups have been integrated into recent clinical guidelines and consensus statements (Grundy et al., 20182019; Cegla et al., 2019; Mach et al., 2019; Wilson et al., 2019; Handelsman et al., 2020; Pearson et al., 2021; Reyes-Soffer et al., 2022). In this article, we review hyper-Lp(a) in the context of FH, particularly in respect of testing for Lp(a) during cascade testing of relatives of probands with a definite diagnosis of FH. We also refer to the impact of elevated Lp(a) on the phenotypic diagnosis of FH.

## Double-Trouble: FH and Hyper-Lp(a) as Dual Risk Factors

### Two Monogenic Defects in Atherogenic Lipoproteins: FH and Hyper-Lp(a)

FH has long been considered a highly penetrant disease, with rarely anyone having a plasma LDL cholesterol (LDL-C) concentration below the 95th percentile of the general population (Miserez and Keller, 1995), however a recent finding that pathogenic or likely pathogenic FH mutations were present even in subjects with LDL<3.3 mmol/L contributing to 27% of total mutation-positive subjects in a large study suggests large heterogeneity in the clinical expression of monogenic FH (Khera et al., 2016; Sniderman et al., 2022). The majority of FH (80%–85%) is caused by mutations of the *LDLR* gene, with over 1700 mutations identified (Iacocca et al., 2018), causing either deficient or defective LDL receptors (Usifo et al., 2012). Other mutations are *APOB* gene missense mutations (5%–10%), gain-of-function mutations in proprotein convertase subtilisin/kexin type 9 (*PCSK9)* (1%), and mutations of *LDLRAP1* (Nordestgaard et al., 2013; Fellin et al., 2015). The inheritance of FH is autosomal-codominant except for *LDLRAP1* which is autosomal recessive (Fellin et al., 2015). The phenotype of FH is severe in cases with deficient or null LDL receptors, but milder in cases with missense mutations in *LDLR* that do not inactivate LDL receptors completely (Khera and Hegele, 2020), or in familial defective *APOB* mutations (Miserez and Keller, 1995). The cumulative exposure of LDL-C in patients with FH begins in fetal life (Brown et al., 1978). Hence the majority of untreated patients with homozygous FH (HoFH) and heterozygous FH (HeFH) develop symptomatic atherosclerotic coronary artery disease (CAD) before 20 years old and 60 years old respectively (Nordestgaard et al., 2013).

Young men with FH have >25-fold increased relative risk of CAD compared with patients without FH (Hopkins, 2017). A recent meta-analysis highlighted that FH was highly prevalent among patients with ASCVD (1 affected individual in 17 patients), an 18-fold higher frequency than in the general population (Hu et al., 2020). Early initiation of cholesterol-lowering treatment can achieve a 10-fold decreased risk of ASCVD in patients with FH (Perez-Calahorra et al., 2019). However, despite lowering LDL-C to target levels, there is still a residual risk in some patients, a particular culprit being elevated plasma Lp(a) levels (Alonso et al., 2014; Vuorio et al., 2017; Rosenson and Goonewardena, 2021).

Although FH and hyper-Lp(a) are both autosomal co-dominantly inherited, the genetics of FH and hyper-Lp(a) differ. The *LDLR* is located on chromosome 19 and *LPA* on chromosome 6. The *LPA* gene is considered to be fully expressed by 2 years of age, adult plasma Lp(a) concentrations achieved by age of 5 years (Wilson et al., 2019; Strandkjær et al., 2021). However, a very recent report has suggested that Lp(a) increases by ≈ 20% from childhood to adulthood in a large cohort of children with mostly a diagnosis of FH (de Boer et al., 2022). Unlike FH, a pure monogenic disorder, elevated Lp(a) levels are consequent on a combination of heritable factors that contribute to >90% of plasma concentrations. Lp(a) concentrations are mainly determined by copy number variation of the Kringle IV type 2 (K-IV_2_) repeats, with over 2000 gene variants in the wider *LPA* gene region (Burgess et al., 2018; Trinder et al., 2020a; Hoekstra et al., 2021; Said et al., 2021), and apoE genotypes (Moriarty et al., 2017). Owing to the inheritance of >40 possible different allelic *LPA* variants, the multiple copies of K-IV_2_ domain of the apo(a) isoform result in a widely variable molecular mass of Lp(a) that contributes up to 1000-fold differences in plasma Lp(a) concentrations (Schmidt et al., 2016). Over 500 gene variants in the K-IV_2_ repeats region have a major effect on Lp(a) concentration (Coassin et al., 2019). The relationship between Lp(a) genetics and concentration varies with ethnicity (Dumitrescu et al., 2011; Paré et al., 2019). Higher Lp(a) concentrations are observed among people of African descent and Indians, whereas lower concentrations are reported in Chinese and Hispanic populations (Enkhmaa et al., 2016; Paré et al., 2019; Loh et al., 2021; Patel et al., 2021). Studies, in general, support the clinical use of Lp(a) rather than apo(a) isoforms or single nucleotide polymorphisms (SNPs) in predicting the risk of ASCVD (Paré et al., 2019; Trinder et al., 2020a; Page et al., 2020). Phenotype, and hence the penetrance of the genetic defect, appears to be more important in influencing ASCVD risk than genotype with both FH (Perez-Calahorra et al., 2019) and elevated Lp(a) levels (Paré et al., 2019; Trinder et al., 2020a).

The risk of ASCVD is directly related to the plasma concentration of Lp(a). An analysis of 460,506 participants from the United Kingdom Biobank database showed that Lp(a) was linearly associated with ASCVD risk after a median follow up of 11.2 years, with a hazard ratio (HR) of 1.11 per 50 nmol/L increments within each ethnicity (White, South Asian, Black), despite ethnic differences in median Lp(a) values (Patel et al., 2021). Lp(a) concentration ≥150 nmol/L (≈70 mg/dL) was associated with increased incidence of CAD in both primary and secondary prevention groups (HR 1.63 and HR 1.23 respectively), with weaker associations being found with ischaemic stroke (Patel et al., 2021). An early meta-analysis by the Emerging Risk Factors Collaboration of 126,634 people without prior history of CAD or stroke at baseline showed a curvilinear relationship between Lp(a) and cardiovascular outcomes, with the highest risk of CAD and ischaemic stroke being seen at extremely high Lp(a) levels (Emerging Risk Factors et al., 2009). Extremely high plasma Lp(a) concentrations (>95th percentile) are associated with the highest risk of ASCVD (Kamstrup et al., 2008; Burgess et al., 2018; Loh et al., 2021), CAVD (Kamstrup et al., 2014; Guddeti et al., 2020), and heart failure (Kamstrup and Nordestgaard, 2016) in the general population. Mendelian randomization studies employing polygenic risk scores suggest that an extremely elevated Lp(a) (>430 nmol/L or >180 mg/dL) is a risk factor for ASCVD equivalent to HeFH (Burgess et al., 2018; Trinder et al., 2020a).

### Is Hyper-Lp(a) More Common in FH?

Plasma Lp(a) concentration above 50 mg/dL (≈100–125 nmol/L), that is above the 80th percentile for a Caucasian population, is commonly accepted in clinical practice as an elevated level (Nordestgaard et al., 2010; Grundy et al., 20182019; Wilson et al., 2019; Pearson et al., 2021), and is used as a clinically meaningful threshold level in many studies. Hyper-Lp(a) is more prevalent in HeFH than in the general population (29.3% vs. 22.2%), as shown in a large study of 2,917 patients with HeFH by (Alonso et al., 2014). The prevalence of hyper-Lp(a) may be as high as 30%–50% of patients with HeFH (Vuorio et al., 2020). Although the genetic control of Lp(a) and FH is a priori orthogonal (Ellis et al., 2019), a defective LDL receptor pathway may contribute to elevating Lp(a) levels. Patients with HoFH due to *LDLR* mutations have been reported to have an almost 2-fold higher Lp(a) level than those with HeFH signifying a gene dosage effect (Kraft et al., 2000), although not all studies are concordant (Sjouke et al., 2017). Also, Lp(a) concentrations were reported to be higher in patients with FH (*LDLR* and *PCSK9* mutations) compared with controls (Tada et al., 2016), and Lp(a) levels are modestly lowered by PCSK9 inhibitors (Bittner et al., 2020; O'Donoghue et al., 2019). Lp(a) levels were numerically higher in FH patients with *LDLR* null mutations compared with those with defective mutations in the SAFEHEART study (Alonso et al., 2014). Although Lp(a) concentrations have been reported to be high in FH, it remains unclear whether this involves decreased Lp(a) clearance via the LDL receptor (Vuorio et al., 2020). Lp(a) levels are not altered or even increased by statin, which upregulates LDL receptors (Tsimikas et al., 2020a). It has been suggested that elevated levels of Lp(a) in FH may be due to ascertainment bias (Trinder et al., 2020b) and population-based studies do not suggest that FH is associated with Lp(a) (Langsted et al., 2016).

### Hyper-Lp(a) as a Risk Enhancer in FH

**FIGURE 1:**
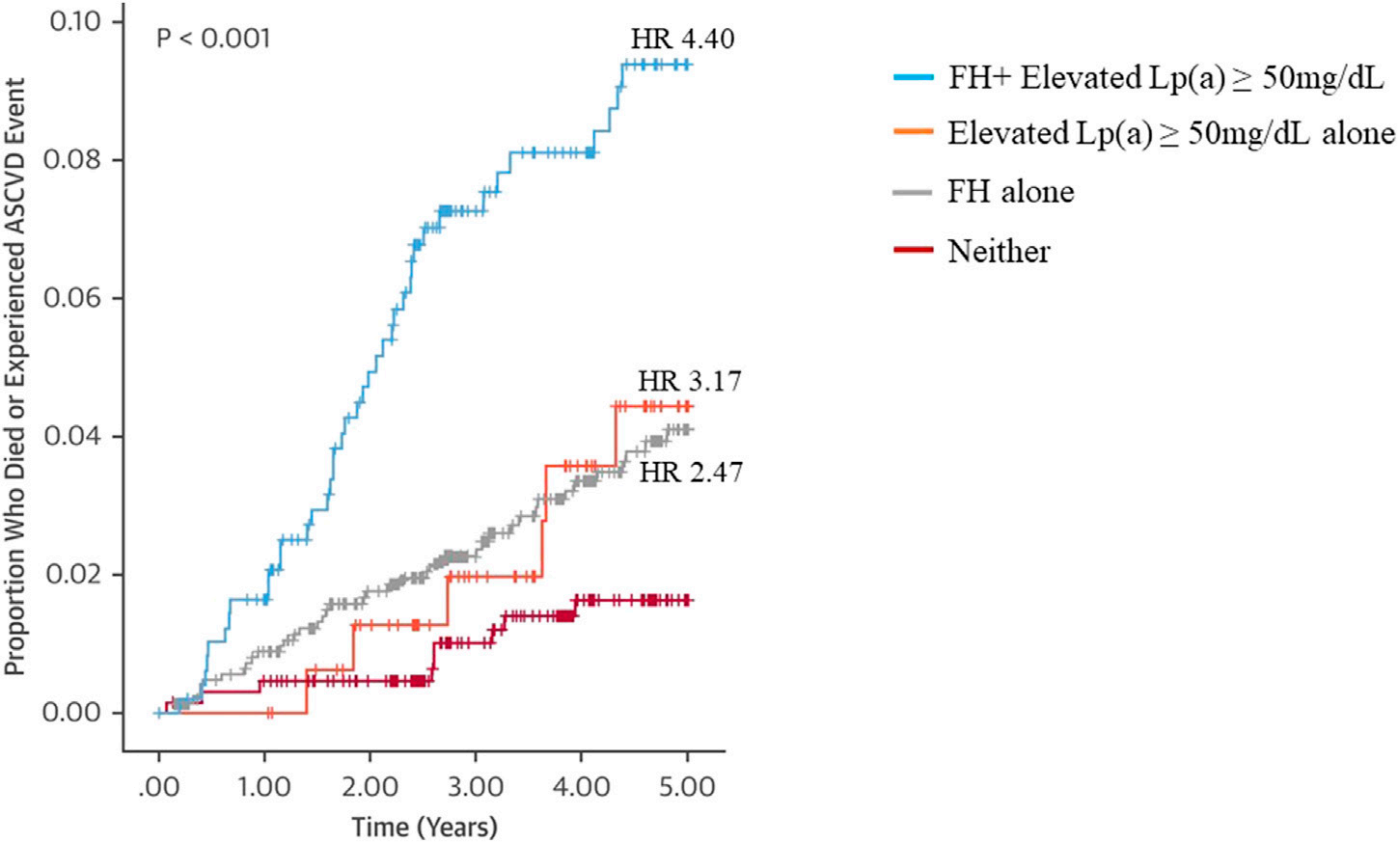
Kaplan-Meier survival analysis showing a significant association between the FH and Lp(a) diagnoses of relatives and ASCVD events. Cox proportional hazard ratio (HR) of patients with dual FH and Lp(a) diagnoses, elevated Lp(a) alone, FH alone compared with individuals with neither disorder are shown in the figure. Modified from Ellis KL et al. Value of Measuring Lipoprotein (a) During Cascade Testing for Familial Hypercholesterolemia. J Am Coll Cardiol. 2019; 73:1029–1039, with permission.

About 10% of patients with HeFH have ASCVD events even after 12 years of high intensity cholesterol-lowering treatment, pointing to significant residual risk (Perez-Calahorra et al., 2019). An important cause is hyper-Lp(a) (Alonso et al., 2014; Vuorio et al., 2017; Rosenson and Goonewardena, 2021; Alonso et al., 2022). Premature ASCVD risk conferred by excessive exposure to LDL-C in patients with FH is further accentuated by elevated Lp(a) levels and this appears to be independent of the type of FH mutation (Alonso et al., 2014; Vuorio et al., 2020). In a Spanish cohort of HeFH patients (SAFEHEART), patients with a combination of null *LDLR* mutation and hyper-Lp(a) > 50 mg/dL had the worst CVD-free survival time compared with patients with non-null *LDLR* mutation and/or Lp(a) < 50 mg/dL (Alonso et al., 2014). The risk of ASCVD or mortality of relatives of patients with FH was also highest in relatives with both FH and hyper-Lp(a) (HR 4.40) than in those with FH alone (HR 2.47), and hyper-Lp(a) alone (HR 3.17) when compared with individuals with neither disorder (Figure 1) (Ellis et al., 2019). These studies suggest that hyper-Lp(a) is an independent residual risk factor for ASCVD in FH. Several other prospective studies have also shown a significant association between elevated Lp(a) levels and increased risk of ASCVD in FH and are summarized in Table 1.

**TABLE 1:** Selected prospective studies in familial hypercholesterolemia showing the association between lipoprotein(a) levels and atherosclerotic cardiovascular disease risk.

Study population, published year	Characteristics, number of subjects (n)		Outcome, follow up (yr)	Hazard ratios (95% confidence interval) for ASCVD risk multivariable regression analysis		p value
Copenhagen General Population Study Langsted et al. (2016)	Phenotypic Diagnosis of FH using modified DLCN, Simon Broome, MEDPED		MI	Neither disorder (reference), *n* = 35153	1	<0.001 (log-rank trend)
	Probable or definite FH	184	Median follow up: 3.9 years	Lp(a) > 50 mg/dL alone, *n* = 6921	1.4 (1.1–1.7)	
	Possible FH	3082		FH alone, *n* = 2300	3.2 (2.5–4.1)	
	Unlikely to have FH	42934		FH and Lp(a) > 50 mg/dL, *n* = 715	5.3 (3.6–7.6)	
		*n* = 46200				
SAFEHEART Pérez de Isla et al. (2017) (Spanish)	Genetic diagnosis (includes patients with and without history of ASCVD) *n* = 2404		ASCVD event or death	Lp(a) ≤50 mg/dL (reference)	1	0.028
			Mean follow up 5.5 years	Lp(a) > 50 mg/dL	1.52 (1.05–2.21)	
SAFEHEART Ellis et al. (2019) (Spanish)	Relatives of probands with genetic diagnosis of FH		ASCVD event or death Mean follow up 3.5 years	Neither disorder (reference), n = 780	1	
	Genetically positive FH	1944		Lp(a) ≥50 mg/dL alone, *n* = 203	3.17 (1.16–8.64)	0.024
	No FH	983		FH alone, *n* = 1413	2.47 (1.06–5.74)	0.036
		*n* = 2927		FH and Lp(a) ≥50 mg/dL, *n* = 531	4.40 (1.92–10.07)	<0.001
Cao et al. (2019) (Chinese)	Phenotypic diagnosis of FH using DLCN score >6 (definite and probable FH) from a patient population with suspected CVD *n* = 393		ASCVD event Mean follow up 3 years	Lp(a) per log unit increase	2.03 (1.28–3.21)	0.002
				Lp(a) Tertile 1 (reference)	1	
				Lp(a) Tertile 2	4.99 (1.36–9.25)	0.015
				Lp(a) Tertile 3	6.96 (2.24–9.32)	0.001
Multicenter study from 5 registries Paquette et al., 2021 (Montreal, British Columbia, United Kingdom Biobank, Ontario, France), 2021	Phenotypic diagnosis of FH (DLCN ≥6 points) or genetic diagnosis (74%), without prior history of ASCVD *n* = 3881		ASCVD Mean follow up 8 years	Lp(a) < 50 mg/dL (reference)	1	0.004
				Lp(a) ≥50 mg/dL	1.53 (1.14–2.04)	

ASCVD, atherosclerotic cardiovascular disease; DLCN, dutch lipid clinic network; FH, familial hypercholesterolemia; Lp(a), lipoprotein (a); MEDPED, make early diagnosis to prevent early death criteria; MI, myocardial infarction.

Reference: refers to this group being the reference group for comparison.

**FIGURE 2:**
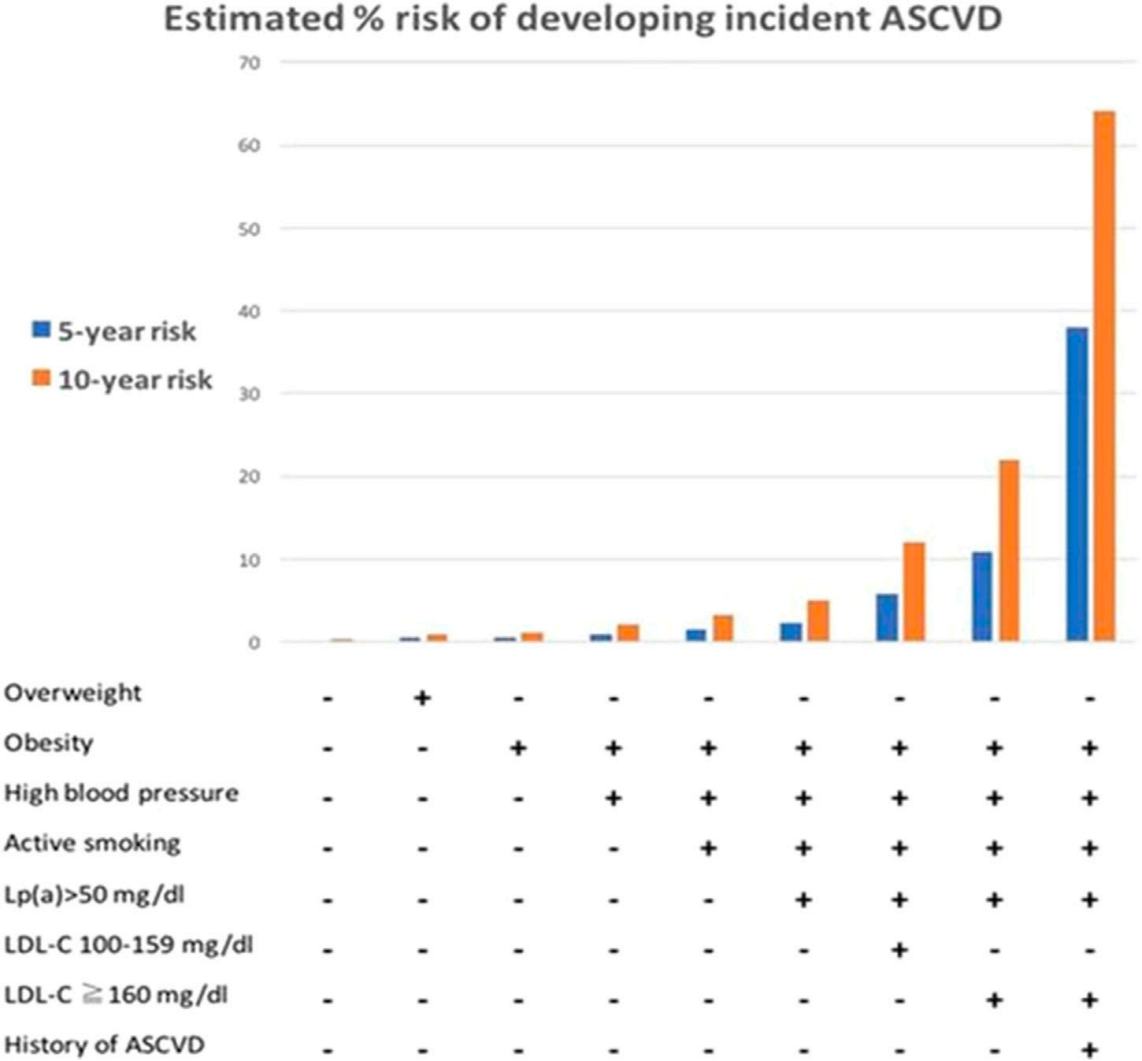
Graphical use of SAFEHEART-RE for predicting 5 and 10-year risks of developing incident atherosclerotic cardiovascular disease (ASCVD) in familial hypercholesterolemia (FH) in primary prevention or secondary prevention settings; an example shown refers to a 66-year-old man with FH and low-density lipoprotein-cholesterol (LDL-C) <100 mg/dl (2.6 mmol/L) in absence or presence of elevated lipoprotein (a) [Lp(a)] > 50 mg/dl. From Perez de Isla L et al. Predicting Cardiovascular Events in Familial Hypercholesterolemia: The SAFEHEART Registry (Spanish Familial Hypercholesterolemia Cohort Study). Circulation. 2017; 135:2133–2144, with permission.

Lp(a) may be useful in improving ASCVD risk prediction in high-risk groups (Grundy et al., 20182019; Wilson et al., 2019; Cegla et al., 2019; Handelsman et al., 2020; Pearson et al., 2021), particularly in FH (Santos et al., 2016; Pérez de Isla et al., 2017; Paquette et al., 2021). The two major risk equations, the SAFEHEART-RE (Pérez de Isla et al., 2017) and the FH-Risk-Score (Paquette et al., 2021), were derived from large prospective FH populations and included hyper-Lp(a) [Lp(a) > 50 mg/dL (Pérez de Isla et al., 2017) or ≥50 mg/dL (Paquette et al., 2021)] as a predictor of ASCVD risk in patients with the FH in primary (Pérez de Isla et al., 2017; Paquette et al., 2021) and secondary prevention settings (Pérez de Isla et al., 2017). Figure 2 shows an example of using the SAFEHEART-RE to predict 5- and 10-year risks of developing incident ASCVD in FH. The SAFEHEART-RE risk equation was based on an exclusively genetically defined population, whereas the FH-Risk-Score was based on a population defined using a mixture of genetic and phenotypic criteria. In the multivariate analyses that formulated these two risk equations, the hyper-Lp(a) variable was associated with similar hazard ratios in both studies; HR 1.5 [SAFEHEART RE: HR1.52 (95% CI 1.05–2.21) (Pérez de Isla et al., 2017), FH-Risk-Score: HR 1.53 (1.14–2.04) (Paquette et al., 2021)], Table 1. In the FH-Risk-Score, the risk attributed by Lp(a) ≥50 mg/dL as a binary variable (yes/no) carried more weightage than untreated LDL-C 5.5–7.5 mmol/L (Paquette et al., 2021). It follows that extremely elevated Lp(a) concentrations (>95 percentiles, 100 mg/dL or 200 nmol/L) would be strongly predictive of future ASCVD in FH. Conversely, low Lp(a) levels (1–74th percentile, 0–30 mg/dL or 0–60 nmol/L) may contribute to resilience against ASCVD in FH (Perez de Isla et al., 2021).

Hyper-Lp(a) is also an independent risk factor for CAVD in both the general population (Kamstrup et al., 2014; Guddeti et al., 2020) and in patients with FH (Vongpromek et al., 2015; Mata et al., 2021). The mechanisms are unclear, but are likely mediated by oxidized phospholipids carried by Lp(a) (Yeang et al., 2016; Kamstrup et al., 2017), with contributions also from the cumulative burden of LDL-C, increasing age, and a history of hypertension (Mata et al., 2021; Pérez de Isla et al., 2021). CAVD itself is a risk factor for ASCVD, heart failure, and mortality, further contributing to residual risk in FH (Yeang et al., 2016; Kamstrup et al., 2017; Mata et al., 2021). The prevalence of aortic valve calcification is two-fold more common in HeFH than in controls (Ten Kate et al., 2015), and is present in 40%–60% of asymptomatic HeFH (Ten Kate et al., 2015; Vongpromek et al., 2015). In a family study of 17 patients with both calcific aortic valve stenosis and Lp(a) ≥60 mg/dL, (Perrot et al., 2019) showed that their first-degree relatives were at increased risk of aortic valve microcalcification and stenosis compared with a control group of normal Lp(a) levels . Although this was a small and underpowered study, the relatively high yield of detection of 48.5% of aortic valve microcalcification suggests that cascade screening of families for CAVD and elevated Lp(a) may be potentially useful (Perrot et al., 2019), but further studies are required.

### Cumulative ASCVD Burden Due to Two Genetic Risk Factors

**FIGURE 3:**
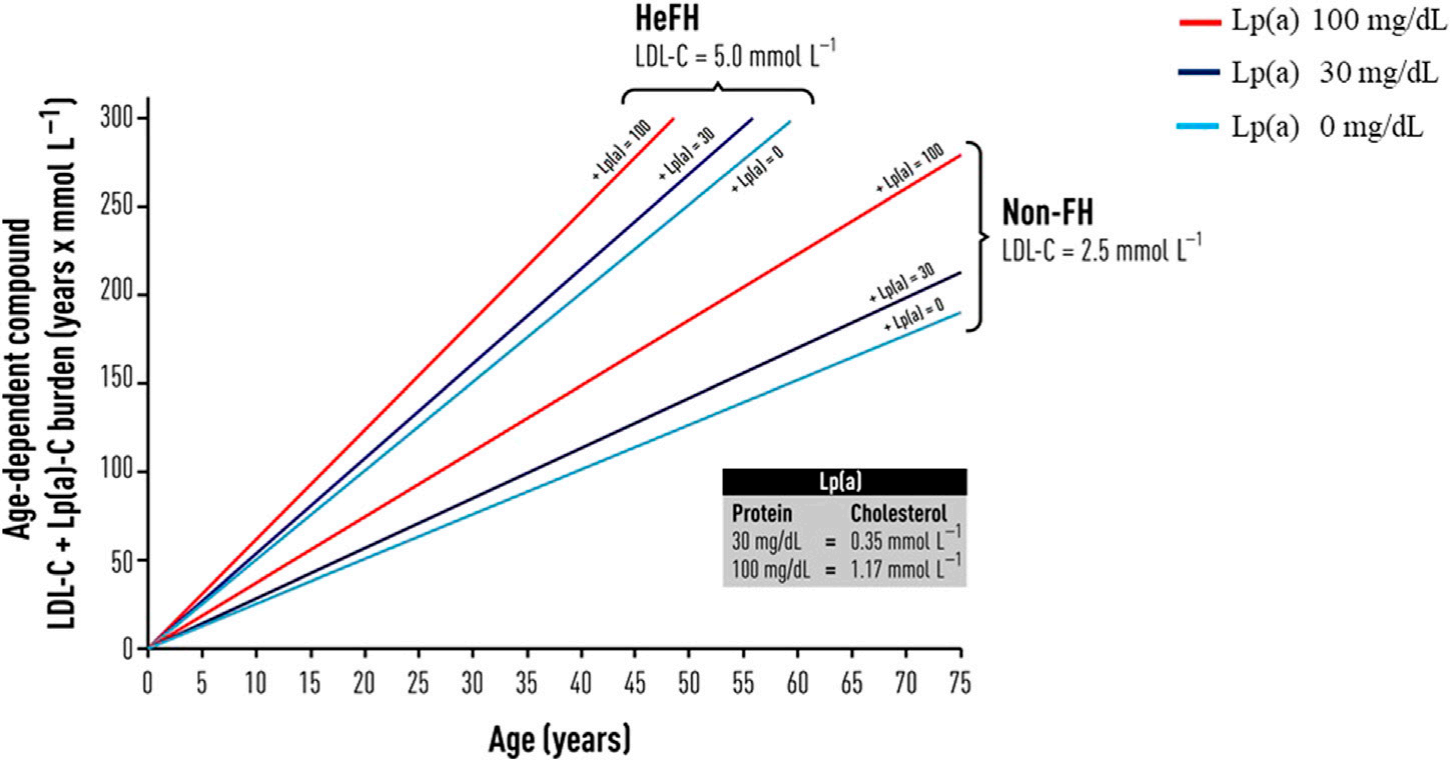
Schematic diagram of the concept of compound LDL- and Lp(a)--cholesterol burden (*y*-axis) that increases with age, in patients with and without FH, with assumption that in untreated heterozygous FH patients (HeFH) and in non-FH individuals. From Vuorio A, Watts GF, Schneider WJ, Tsimikas S, Kovanen PT. Familial hypercholesterolemia and elevated lipoprotein (a): double heritable risk and new therapeutic opportunities. J. Intern. Med. 2020; 287:2–18, with permission.

Although governed by different genetic pathways, the cumulative effects of these two pro-atherogenic risk factors are more than additive. The lifetime cumulative effect of these dual heritable risk factors was recently illustrated by (Vuorio et al., 2017) using the concept of compound LDL-C and Lp(a)-particle cholesterol burden (Figure 3). Using the combination of the burden of LDL-C-years and Lp(a)-cholesterol years in FH (Vuorio et al., 2017), an earlier onset of CVD burden of 4 years was estimated in patients with untreated HeFH, comparing patients with Lp(a) levels of 100 mg/dL with patients with levels of 30 mg/dL (Vuorio et al., 2020). Because of the significant contribution to ASCVD and CAVD risk, Lp(a) concentration should be measured on at least one occasion as a priority in patients with FH (Grundy et al., 20182019; Cegla et al., 2019; Wilson et al., 2019; Langsted and Nordestgaard, 2022).

## Incorporating Lp(a) in Screening for FH

### Cascade Testing for FH

Despite much guidance on best practices for the care of FH, FH remains mainly underdiagnosed (Nordestgaard et al., 2013). One in 250 people has HeFH, while 1 in 300,000 people has HoFH (Nordestgaard et al., 2013; Watts et al., 2015; Akioyamen et al., 2017; Berberich and Hegele, 2019; Hu et al., 2020; Khera and Hegele, 2020). The prevalence of HeFH may be up to 1% in gene founder populations and up to 20% in patients with premature ASCVD (De Backer et al., 2015; Berberich and Hegele, 2019). The Center for Disease Control and Prevention recommends cascade testing for FH (the leading and actionable Tier condition), because early diagnosis can be aided by genetic testing, and implementation of ASCVD prevention measures can have a significant public health impact. Cascade testing for FH has been shown to be highly cost-effective in multiple populations (Marang-van de Mheen et al., 2002; Ademi et al., 2013; Kerr et al., 2017; Knowles et al., 2017; Lázaro et al., 2017), including children (Wald et al., 2016; Ademi et al., 2020; Jackson et al., 2021). A related approach is child-parent testing, following the detection of a child at immunization at 2 years old (Wald et al., 2016). Ademi et al. (2020)recently showed that cascade screening in ten-year-old children followed by early initiation of statin in children with HeFH is cost-effective compared with usual care, with a predicted significant lifetime reduction in hospitalization costs. In this study, the prevalence of a positive FH mutation among children detected through cascade screening was 56.8% (Ademi et al., 2020).

### Cascade Testing for Both FH and Hyper-Lp(a)

**FIGURE 4:**
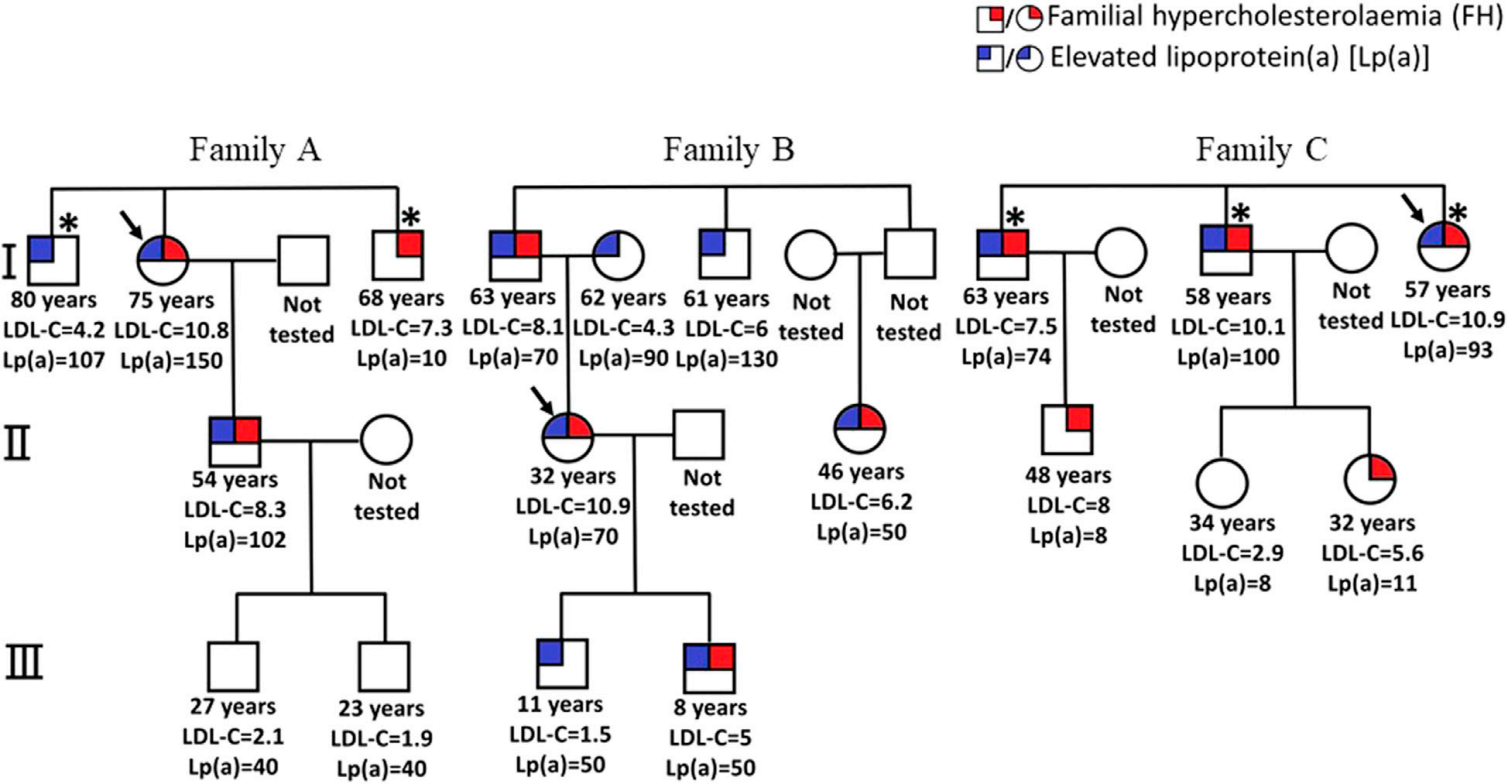
Pedigrees of families (A–C) with familial hypercholesterolemia (FH) and elevated lipoprotein (a) [Lp(a)] ≥ 50 mg/dL, with their LDL cholesterol concentrations (mmol/L) and Lp(a) concentrations (mg/dl). In families (A,C), two new cases of FH and elevated Lp(a) were identified among five relatives tested. In family (B), all six relatives tested were found to have elevated Lp(a) (i.e., one new case of elevated Lp(a) for every relative tested) and three identified with FH. However, there were families where fewer, or no relatives were detected than those shown in these three families. Asterisk * indicates coronary artery disease. From Chakraborty A et al. Cascade testing for elevated lipoprotein (a) in relatives of probands with familial hypercholesterolemia and elevated lipoprotein(a). Atherosclerosis 2021, with permission.

The distribution of Lp(a) in the general population is positively skewed to the right, with hyper-Lp(a) coinciding with the top 20th percentile of the population distribution (Nordestgaard et al., 2010). This equates to 1 in 5 people having hyper-Lp(a) in the general population, although studies have shown that there are ethnic differences in prevalence (Paré et al., 2019; Patel et al., 2021). It is estimated that worldwide hyper-Lp(a) is a common disorder affecting 1.4 billion people and that at least five million have both HeFH and hyper-Lp(a) (Vuorio et al., 2020). Hyper-Lp(a) is, therefore, more common than HeFH and may be more prevalent in patients with HeFH (Alonso et al., 2014) than those without. Cascade testing from affected probands with FH or high Lp(a) levels is useful in identifying these conditions. Figure 4 shows examples of the pedigrees of three families who were cascade tested for FH and high Lp(a).

**FIGURE 5:**
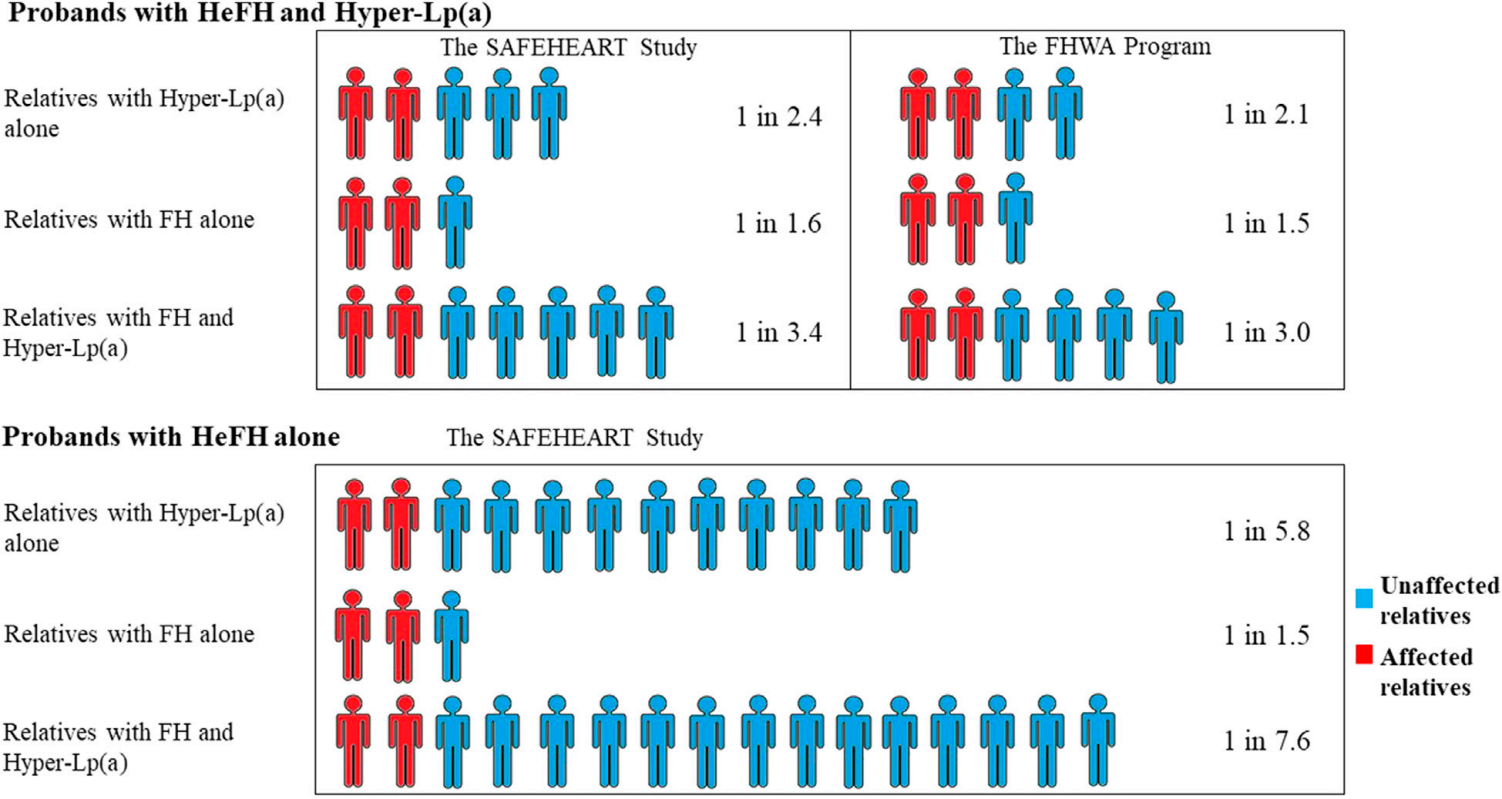
Yield of detection of diagnoses of elevated lipoprotein (a) [Lp(a)] and familial hypercholesterolemia (FH) during cascade screening of relatives from HeFH probands with or without hyper-Lp(a). One individual of hyper-Lp(a) without FH was detected for every 2.1–2.4 relatives screened if the proband had HeFH and hyper-Lp(a), whereas the yield of detection was one individual for every 5.8 relatives screened if the proband had HeFH but not hyper-Lp(a). HyperLp(a) refers to Lp(a)≥ 50 mg/dL. The SAFEHEART Study by Ellis et al., (2019); The FHWA Program by Chakraborty et al., (2021).

Two major studies showed that incorporating Lp(a) into the genetic cascade testing of FH in families of probands with FH mutations is an effective way of identifying hyper-Lp(a) in this high-risk group (Ellis et al., 2019; Chakraborty et al., 2021). In these two studies, the yield of detection of FH *via* cascade screening was 1 individual for every 1.5–1.6 relatives screened. The yield of detection of hyper-Lp(a) in FH cascade screening was also high: 1 individual of hyper-Lp(a) was detected for every 2.1–2.4 relatives screened, whereas the yield of detection of both hyper-Lp(a) and FH was 1 individual for every 3–3.4 relatives screened (Figures 5). Unsurprisingly, screening from probands with FH and hyper-Lp(a) levels compared with screening from probands without hyper-Lp(a) had a higher yield of detection of relatives with hyper-Lp(a) and FH (1 in 3.4 vs. 1 in 7.6) (Ellis et al., 2019). With FH probands in absence of hyper-Lp(a), the yield of detection of hyper-Lp(a) among relatives (1 individual in 5.8 relatives) was similar to the general population (1 in 5 people) (Nordestgaard et al., 2010). Ellis et al. (2019) also showed that the yield of detection of hyper-Lp(a) decreases with increasing generational separation from the proband with hyper-Lp(a): 1 individual in 2 of 1st degree relatives screened have hyper-Lp(a), 1 individual in 2.9 of 2nd degree relatives, and 1 in 3.3 of 3rd degree relatives. Concordance of detection of FH and Lp(a) was low and non-significant, implying that the conditions are genetically orthogonal.

### Cascade Testing for Hyper-Lp(a) Alone

The autosomal co-dominant heritability of Lp(a) is confirmed by pedigree studies (Utermann et al., 1987; Genest et al., 1991). Genest et al. (1991) showed that Lp(a) levels were significantly correlated between parents-and-offsprings, whereas Lp(a) levels of proband-and-spouse were not. Studies correlating Lp(a) levels of the younger generation and family history showed that the serum Lp(a) levels of the offspring were significantly and directly associated with a positive CAD history of parents (Kostner et al., 1991; Srinivasan et al., 1991; Routi et al., 1996), but less so with CAD history in second- and third-degree relatives; higher Lp(a) levels in children were associated with a positive CAD history in their grandparents in one study (Wilcken et al., 1993) but not another. (Routi et al., 1996).

Small apo(a) isoforms are associated with higher Lp(a) levels (Kronenberg, 2016). The alleles of genes determining the apo(a) isoform size are randomly inherited from each parent and the expression of the inherited alleles is dominated by the alleles controlling small apo(a) isoform size (high Lp(a) levels) (Kronenberg, 2016). This explains the observation that an offspring with extremely high Lp(a) level may have both or one parent with elevated Lp(a). Children from parents with low Lp(a) levels have low Lp(a) levels, unless there is a secondary cause for elevated Lp(a). When only 1 out of 2 parents has elevated Lp(a), the Lp(a) level of the offspring will on average range from normal to moderately elevated in most instance (Genest et al., 1991).

Non-genetic factors should be considered if the Lp(a) levels of the offspring differ greatly from parents. This also applies to testing adult relatives of the proband with high Lp(a). Acquired causes that could markedly elevate Lp(a) level (up to 3-fold) include nephrotic syndrome, severe renal impairment, use of growth hormones, and pregnancy, while Lp(a) levels may be moderately elevated by 20%–50% from hypothyroidism, menopause, inflammatory conditions, and testosterone deficiency (Kostner et al., 2013). Other causes that markedly lowered Lp(a) include cholestatic liver disease (up to 90% reduction) (Chennamsetty et al., 2011), whereas Lp(a) levels are moderately lowered by PCSK9 inhibition, niacin, post-menopausal hormone replacement therapy (25% reduction) (O'Donoghue et al., 2019), as well as mildly lowered by other medications e.g. fibrates, aspirin, angiotensin-converting enzyme inhibitor, calcium antagonist (<10% reduction) (Nordestgaard et al., 2010). Owing to a recent observation that Lp(a) may rise with age, especially in children with suspected FH, Lp(a) level should be rechecked in adulthood (de Boer et al., 2022). A probable increase in Lp(a) may relate to the effects of age, endocrine changes, use of statin, and other secondary factors (de Boer et al., 2022).

## Proposed Management of Elevated Lp(a): Lessons From Models of Care for FH

**FIGURE 6:**
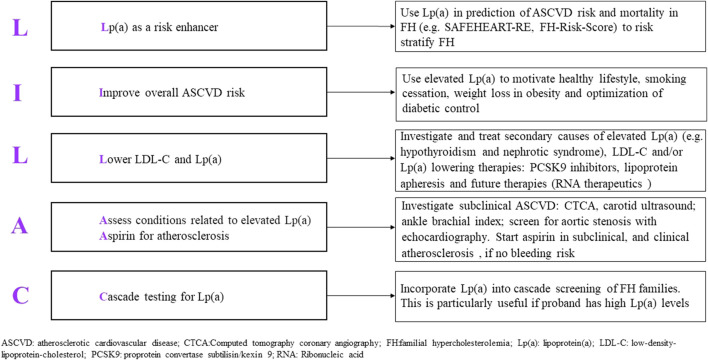
Proposed mnemonic (LILAC) for the management of elevated lipoprotein (a) in familial hypercholesterolemia.

**FIGURE 7:**
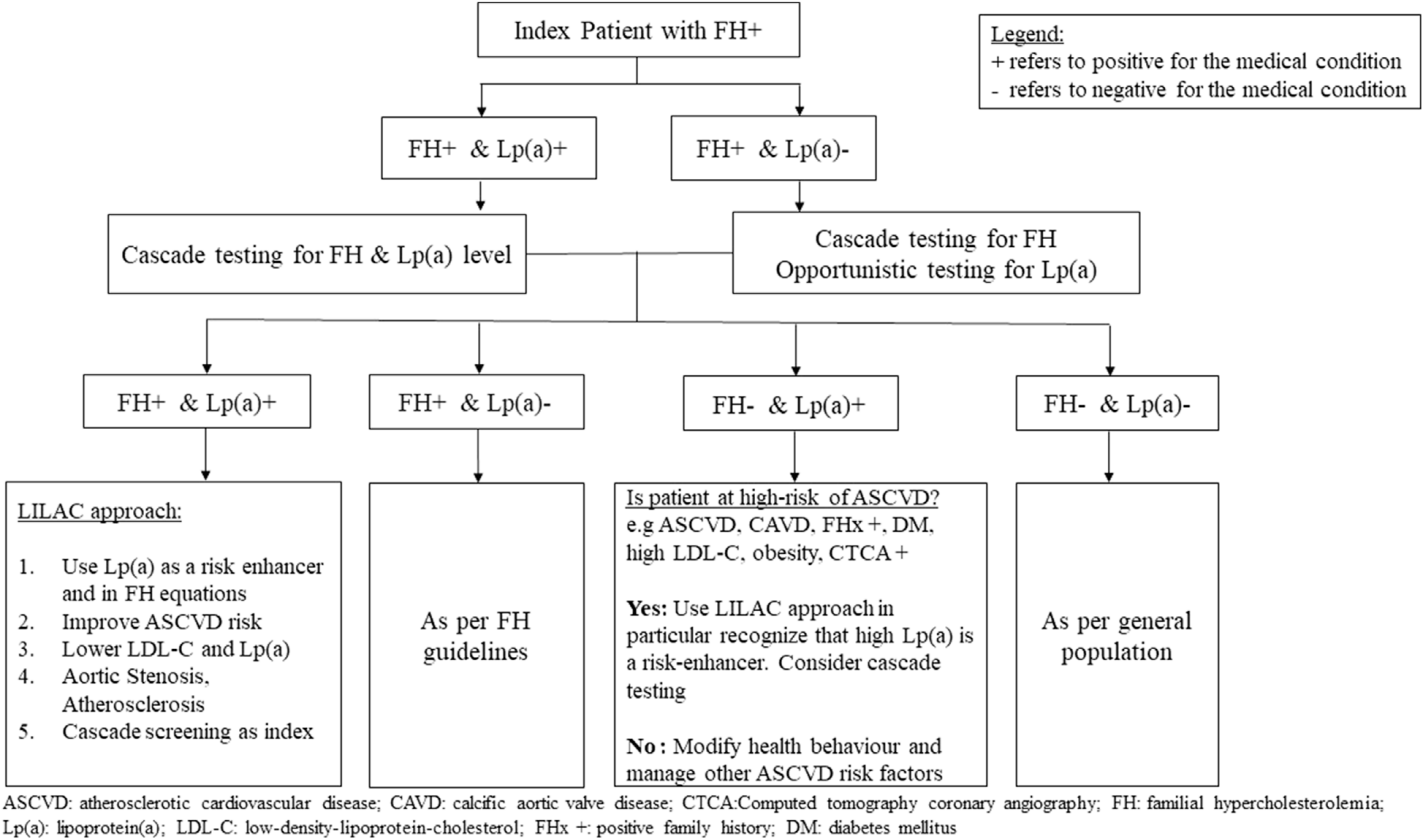
Proposed algorithm for the management of relatives after cascade testing for hyper-lipoprotein (a) from probands with familial hypercholesterolemia.

Assessment of Lp(a) is valuable in stratifying the risk of ASCVD in FH (Thanassoulis, 2019; Tsimikas and Stroes, 2020; Watts et al., 2020). We propose the principles of management of patients with elevated Lp(a) according to five salient points, guided by the mnemonic LILAC (Figure 6). Starting with detection, Lp(a) should be tested in all patients with FH as this is a necessary step in the risk stratification of ASCVD in FH. Hyper-Lp(a) is defined as ≥50 mg/dL (≈100–125 nmol/L) (Grundy et al., 20182019; Wilson et al., 2019; Pearson et al., 2021), noting that patients with extremely high Lp(a) levels (>150 mg/dL or 300 nmol/L) have the highest risk. Management of hyper-Lp(a) includes a thorough history taking of family and personal history of premature ASCVD, as well as identifying early signs of ASCVD. Knowledge of Lp(a) can be utilized in these specific risk equations for ASCVD (Pérez de Isla et al., 2017; Paquette et al., 2021). Blood testing should exclude secondary causes of elevated Lp(a), such as hypothyroidism, kidney impairment, proteinuria, and cholestatic liver disease. Investigation for subclinical atherosclerosis e.g., CT coronary calcium score may be considered because this can enable decisions to intensity treatment plans (Santos et al., 2016; Gallo et al., 2021). In FH with high Lp(a) levels, an echocardiogram to confirm calcific aortic valve stenosis at an appropriate age is essential. Treatment for modifiable risk factors such diabetes mellitus, reducing obesity, alcohol, and smoking cessation, should be optimized. Healthy lifestyle, heart-healthy diet, and improving modifiable cardiovascular risk factors should be emphasized in patients with FH, as well as in patients with hyper-Lp(a) as this has significant cardiovascular benefit even in the absence of effective Lp(a) lowering treatment (Figure 7) (Perrot et al., 2017).

There is currently a lack of effective Lp(a) lowering therapies, and thus a lack of concrete evidence yet showing that specific Lp(a)-lowering can reduce ASCVD risk and mortality. This evidently applies to FH. The HORIZON study is a cardiovascular outcome trial of the antisense oligonucleotide APO(a)-L_RX_ (TQJ230) that targets the mRNA transcript of the *LPA* gene and can lower Lp(a) by 80% (NCT04023552) (Tsimikas et al., 2020b). A phase 2 study of GalNac3-conjugated-siRNA (Olpasiran) is also undergoing (NCT04270760) will proceed to the clinical outcome trial. While we await these studies, the effect of Lp(a) lowering onto ASCVD is estimated to be significant. Mendelian randomization analyses suggest that Lp(a) lowering of approximately a 65 mg/dL (Lamina et al., 2019) or 100 mg/dL (Emerging Risk Factors et al., 2009) (i.e., >150–200 nmol/dL) is required to achieve to CAD risk reduction equivalent of 1 mmol/L LDL-C lowering. From Danish population-based studies, the authors of another study estimated that an Lp(a) lowering of 50 mg/dL (105 nmol/L) may be needed to reduce CVD risk by 20% in a secondary prevention setting (Madsen et al., 2020).

However, it is likely that even smaller sustained reductions of Lp(a) level would confer significant clinical benefit in ASCVD risk reductions in patients with established ASCVD, as suggested by post-hoc analyses of PCSK9 inhibitor clinical outcome trials (O'Donoghue et al., 2019; Bittner et al., 2020) and lipid apheresis studies (Jaeger et al., 2009; Leebmann et al., 2013; Waldmann and Parhofer, 2016). PCSK9 monoclonal inhibitors have Lp(a) lowering effect of ≈25%. (O'Donoghue et al., 2019; Bittner et al., 2020). In the FOURIER study, investigating evolocumab versus placebo in patients with ASCVD, it was estimated that reduction of Lp(a) level of 34 nmol/L (95% CI 18.5–97 nmol/L) is associated with a relative risk reduction of 20% of cardiovascular events (O'Donoghue et al., 2019). Although lipid apheresis reduces Lp(a) immediately by 60–75%, this effect is not sustained, with the mean inter-apheresis reduction of Lp(a) being approximately 35% for weekly apheresis (Waldmann and Parhofer, 2016). Studies show that patients with elevated Lp(a) who underwent weekly lipid apheresis have a reduced risk of major coronary events through its Lp(a) lowering effect, even in patients who achieve target LDL-C levels (Jaeger et al., 2009; Leebmann et al., 2013).

## Implications of Recent Studies on Lipoprotein (a): Familial Hypercholesterolemia and Beyond

In patients with very high Lp(a) levels, Lp(a) cholesterol may contribute to 30%–45% of the measured LDL-C (Yeang et al., 2015). Whilst correction of LDL-C concentrations for the cholesterol content of Lp(a) remains contentious, it is clear that in patients with very high Lp(a) concentrations, the cholesterol content of this lipoprotein particle may confound estimation of LDL-C and hence lead to a false positive clinical diagnosis of FH (Chan et al., 2019). This could result in unnecessary DNA testing in individuals with DLCN scores >5. Clinical and population studies have shown that up to 25% of patients with probable/definite FH can be reclassified by adjusting LDL-C for the cholesterol content of Lp(a) (Langsted et al., 2016; Chan et al., 2019). We have previously demonstrated that the diagnostic accuracy of DLCN and SB criteria in predicting the detection of a pathogenic mutation of FH is reduced when Lp(a) level is very high, and that adjusting LDL-C for Lp(a) cholesterol using a correction factor of 30% greatly improved the specificity of DLCN and SB criteria when Lp(a) > 100 mg/dL except when LDL-C was as high as 6.5 mmol/L or more (Chan et al., 2019). It is clinically relevant to clarify the diagnosis of clinical HeFH because patients with dual diagnosis of clinical HeFH and hyper-Lp(a) have the highest risk of myocardial infarction compared with patients with either diagnosis alone (Langsted et al., 2016) (Table 1). The effect of Lp(a) cholesterol on the measured LDL-C also has important clinical implications in achieving the target “LDL-C” in patients irrespective of FH status. However, correction factors use assumptions that a certain percentage of cholesterol makes up Lp(a) mass which can range from 30% – 45%; there are so far no consensus on the usage of correction factor(s) to adjust LDL-C for Lp(a) cholesterol content in clinical practice. Quantification of Lp(a) cholesterol with a recently described assay, which allows for more accurate estimates of true LDL-C, may be recommended over the use of correction factors (Yeang et al., 2021; Yeang et al., 2022).

Studies of cascade testing for high Lp(a) within the context of genetic cascade testing for FH provide proof of principle for the effectiveness of this method for screening for elevated Lp(a) (Ellis et al., 2019; Chakraborty et al., 2021). Cascade testing can identify affected family members with varying risks of ASCVD. Cascade testing for Lp(a) does not require genetic testing for Lp(a) variants or use of a polygenic score (Ellis et al., 2019; Chakraborty et al., 2021). The data also suggest that the yield of identifying relatives with high Lp(a) is directly proportional to the level of Lp(a) in the proband (Ellis et al., 2019; Chakraborty et al., 2021), providing a guide to the design of effective implementation strategies. However, formal health economic evaluations that focus not only on cost-effectiveness, but also on cost-utility analyses are recommended. Studies of patient-reported outcomes and experience measures are also needed. The estimation of ASCVD risk in both probands and affected relatives employing the outcome of measuring Lp(a) can be carried out using a recently validated risk equation (Gallo et al., 2020). The importance of measuring Lp(a) in all patients with FH also relates to predicting increased risk of calcific aortic valve stenosis, which is also related to LDL-C burden and history of hypertension (Pérez de Isla et al., 2021).

While cascade testing for elevated Lp(a), particularly in the context of FH, is likely to be cost-effective, identification of affected probands without FH is essential to trigger the cascade testing of relatives. Universal testing of the population for markedly elevated Lp(a) concentrations above 180 mg/dL (>430 nmol/L) has been promulgated by certain guidelines (Mach et al., 2019; Pearson et al., 2021). An alternative, and not mutually exclusive approach, is to consider the screening of adults with established ASCVD, particularly when testing may lead to the initiation or a change in therapy (e.g., statin, aspirin, antihypertensive treatment), as well as when Lp(a) is being used to restratify ASCVD in people with the following conditions: family history of premature ASCVD (Grundy et al., 20182019; Cegla et al., 2019; Mach et al., 2019; Wilson et al., 2019; Handelsman et al., 2020; Pearson et al., 2021), family history of high Lp(a) (Cegla et al., 2019; Wilson et al., 2019; Handelsman et al., 2020), FH (Grundy et al., 20182019; Cegla et al., 2019; Mach et al., 2019; Wilson et al., 2019; Pearson et al., 2021; Watts et al., 2021), diabetes mellitus, renal impairment or family history of thrombophilia (Tsimikas et al., 2018). Measurement of Lp(a) should also be considered in patients at intermediate or borderline risk of ASCVD, particularly when the decision to initiate cholesterol-lowering therapy remains undecided (Cegla et al., 2019; Mach et al., 2019; Wilson et al., 2019; Pearson et al., 2021). Testing for Lp(a) should also be considered in patients with suboptimal reduction in LDL-C despite good adherence to statin therapy, as well as in those with a history of progressive CAVD (Cegla et al., 2019; Wilson et al., 2019; Handelsman et al., 2020). This recommendation applies mainly to adults, although consideration may also be given to testing children or adolescents with FH with premature ischemic stroke or within the context of cascade testing for high Lp(a) and FH (Wilson et al., 2021).

Clinical use of Lp(a) has to be seen in the context of other cardiovascular risk factors and, hence, the estimation of an individual’s absolute cardiovascular risk in individuals. This implies that health economic evaluations are most likely to be favorable when background risk is increased, as in the presence of established or recurrent ASCVD, FH, diabetes, or multiple cardiovascular risk factors. This notion requires verification.

The detection of Lp(a) seems to be to meet most, but not all, classical criteria for screening for a condition (Wilson et al., 1968). The important ones that are not met are that, with the exception of lipoprotein apheresis, there is no accepted or specific treatment for elevated Lp(a) and hence no agreed policy on who should be treated, noting that policy also depends on a favorable health economic evaluation. Amongst FH patients, the case for offering specific treatment with lipoprotein apheresis relates to is those with recurrent ASCVD and evidence of progressive CAVD. Lowering of Lp(a) with PCSK9 inhibitors has been shown to contribute to a reduction in ASCVD events in post-ACS and high-risk patients (Bittner et al., 2020; O'Donoghue et al., 2019), but trials have not been undertaken specifically in patients with FH. Future studies should address the potential impact of additional therapies that can lower Lp(a), including obicetrapib (a CETP inhibitor) (Hovingh et al., 2015) and resmetirom (a selective thyroid hormone receptor-β agonist) (Harrison et al., 2019). Ongoing studies with RNA based therapies will establish whether specific lowering of Lp(a) of the order of 80% (Tsimikas et al., 2020b), or a mean reduction of 100 mg/dL (Burgess et al., 2018), confers improvement in cardiovascular outcomes in high risk individuals, but none of these trials are specifically being carried out in patients with FH.

## Conclusion

### Towards Implementation of Improved Care for Hyper-Lp(a)

There is clear evidence that elevated Lp(a) is a causal risk factor for ASCVD, CAVD, and cardiovascular and all-cause mortality in both genders and multiple ethnic groups. This risk appears to be particularly markedly increased in patients with FH, in whom the case for cascade testing for elevated Lp(a) extends to the benefit of relatives affected with FH and not affected with FH.

The phenotypic diagnosis of FH may be confounded by the cholesterol content of Lp(a), particularly when this is extremely high and this should be accounted (Langsted et al., 2016; Chan et al., 2019); adjusting for the cholesterol content of Lp(a) is also important when phenotypically cascade testing relatives based on age, gender, and LDL-C levels. Lp(a) is a key component of risk stratification equations in FH (Pérez de Isla et al., 2017; Paquette et al., 2021). A positive association has been reported between coronary artery calcium score and Lp(a) in population studies among people who have not yet developed a coronary event (Gallo et al., 2021). The value of measuring Lp(a) in predicting the risk of aortic stenosis in FH is critically important, particularly so in patients with homozygous FH (Ten Kate et al., 2015; Vongpromek et al., 2015; Pérez de Isla et al., 2021). At present, reduction of Lp(a) in people with FH relies on the use of PCSK9 inhibitors (Bittner et al., 2020; O'Donoghue et al., 2019), noting that niacin is not recommended in European countries. Lipoprotein apheresis is another modality but is expensive, labor-intensive and not widely available. (Waldmann and Parhofer, 2016). Apheresis is reimbursable, however, in certain countries for those with progressive coronary disease and elevated Lp(a) concentration (Leebmann et al., 2013). Antisense oligonucleotide and small interfering RNA approaches are currently being trialed and could in the future be the treatment of choice for patients with elevated Lp(a) and established ASCVD or FH.

While the results of large clinical outcome trials are awaited to fully establish the value of screening for high Lp(a), the use of universal, opportunistic, and selective screening strategies can be enabled by the use of implementation strategies. These are relevant to patients and families with or without FH. Using an isoform independent immunoassay is essential for accurate measurement of Lp(a) (Tsimikas et al., 2018) and this should be a reimbursable test (Pearson et al., 2021). Alerts and interpretive comments on laboratory reports are used to acknowledge the value of high Lp(a) in cardiovascular risk assessment and consideration of referral to or discussion with a specialist. Electronic health records should employ a specific ICD code for elevated Lp(a) which may be utilized to trigger the testing of Lp(a) in clinical practice, as well as linkage studies and economic valuations. The management of Lp(a) at present requires wider promotion and acceptance by practitioners who should develop skills in the clinical use of the test and in shared decision-making concerning risk assessment, cascade testing of first-degree relatives, and modification of behavioral and standard cardiovascular risk factors.

In conclusion, evolving models of care for FH have important lessons that can inform models of care for patients with elevated Lp(a) and other risk factors other than FH, including familial combined hyperlipidemia, diabetes mellitus, and hypertension (Watts et al., 2020). Finally, it is worth emphasizing that most studies to date have been carried out in white, Caucasian populations and further investigation of the significance and management of Lp(a) are needed in other ethnic groups, including black Africans and Southern and Eastern Asians (Tsimikas et al., 2018; Paré et al., 2019).
